# Impact of Childhood Maltreatment on the Recognition of Facial Expressions of Emotions

**DOI:** 10.1371/journal.pone.0141732

**Published:** 2015-10-28

**Authors:** Martina Ardizzi, Francesca Martini, Maria Alessandra Umiltà, Valentina Evangelista, Roberto Ravera, Vittorio Gallese

**Affiliations:** 1 Department of Neuroscience, University of Parma, Parma, Italy; 2 Ravera Children Rehabilitation Centre (RCRC), Lakka, Freetown, Sierra Leone; 3 Department of Health Psychology, Hospital of Sanremo, Sanremo, Italy; 4 Department of Pharmacy, University of Parma, Parma, Italy; University of Udine, ITALY

## Abstract

The development of the explicit recognition of facial expressions of emotions can be affected by childhood maltreatment experiences. A previous study demonstrated the existence of an explicit recognition bias for angry facial expressions among a population of adolescent Sierra Leonean street-boys exposed to high levels of maltreatment. In the present study, the recognition bias for angry facial expressions was investigated in a younger population of street-children and age-matched controls. Participants performed a forced-choice facial expressions recognition task. Recognition bias was measured as participants’ tendency to over-attribute anger label to other negative facial expressions. Participants’ heart rate was assessed and related to their behavioral performance, as index of their stress-related physiological responses. Results demonstrated the presence of a recognition bias for angry facial expressions among street-children, also pinpointing a similar, although significantly less pronounced, tendency among controls. Participants’ performance was controlled for age, cognitive and educational levels and for naming skills. None of these variables influenced the recognition bias for angry facial expressions. Differently, a significant effect of heart rate on participants’ tendency to use anger label was evidenced. Taken together, these results suggest that childhood exposure to maltreatment experiences amplifies children’s “pre-existing bias” for anger labeling in forced-choice emotion recognition task. Moreover, they strengthen the thesis according to which the recognition bias for angry facial expressions is a manifestation of a functional adaptive mechanism that tunes victim’s perceptive and attentive focus on salient environmental social stimuli.

## Introduction

Infancy and childhood are periods characterized by significant advances in social and emotional development [1]. For example, the explicit recognition of facial expressions of emotions starts early in infancy and continues through childhood to adolescence and adulthood [2–6]. Children first categorize expressions into superordinate categories of joy and non-joy, and progressively, they distinguish the subordinate categories of negative facial expressions, with anger recognized first [2,7]. Several studies demonstrated that the normal development of the explicit recognition of emotions can be influenced by childhood maltreatment experiences defined as “any act of omission or commission that results in harm or the potential for harm, regardless of intent” [8]. In a recent review it has been highlighted that maltreated children tended to exhibit an overall impairment in facial expression recognition and a greater reactivity, a response bias and a selective electrophysiological activation of specific brain areas in response to faces expressing negative emotions, especially anger [9]. Particularly interesting for the present study is the response bias showed by maltreated children in favor of angry facial expression thanks to which this facial expression is recognized basing on less sensory inputs [10] and fewer expressive cues [11] than other negative facial expressions of emotions. This phenomenon has been interpreted as a specific form of experiential learning by which victims adapt their pre-existing perceptual and attentive mechanisms to process environmental aspects which become especially salient [12,13].

Generally, empirical studies focus primarily on a single type of childhood maltreatment experience (e.g., physical abuse or neglect), recruiting different samples of victims (e.g., victims of physical intra-familiar abuse or post-institutionalized children). This methodological approach allows to understand the specific impact of different maltreatment experiences on the investigated process. However, a sharp distinction between different types of maltreatment conditions appears to be more artificial than real. For example, episodes of physical abuse occur among institutionalized population, as well as, neglect conditions can be lived by physically abused children. Sure enough, abusive parents show less positive and more negative emotions than non-abusive parents [14,15] but also they tend to isolate themselves and their children from interactions with others [16]. On the other hand, institutional care is characterized by both psychosocial deprivation, due to elevated child-to-caregiver ratio, and high peer-competition [17,18]. From this point of view, street-boys’ life conditions represent an exemplificative and extreme case of concurring conditions of abuse and neglect, which come under the more extensive concept of child maltreatment [8]. Street-boys are defined as “Any girl or boy … for whom the street (in the widest sense of the word, including unoccupied dwellings, wasteland, etc.) has become his or her habitual abode and/or source of livelihood; and who is inadequately protected, supervised, or directed by responsible adults” [19]. Street-boys have limited access to basic resources (e.g., adequate food, shelter, clothing, medical care) and they act and suffer high levels of violence, intimidation, robberies, and sexual or physical assaults in the street [20]. Thus, street-boys are exposed to repetitive and protracted experiences of physical abuse and neglect, arose outside familiar environment and exacerbated by the absolute lack of any significant adult-care. A previous study [21] investigated the explicit recognition of facial expressions of emotions in an adolescent population of street-boys (mean age: 15.7 years), demonstrating that the exposure to high levels of maltreatment caused the well-known recognition bias favoring anger to the detriment of fear and sadness recognition. Furthermore, a deep alteration of physiological responses to facial expressions of emotions was established.

It is important to note that when addressing childhood maltreatment, victims’ age plays a fundamental role, because it determines the level of development with which the negative event interferes [22]. This assumption becomes especially significant considering that the explicit recognition of facial expressions of emotions is a social competence that improves during childhood [2]. Furthermore, in a recent review of the literature about researches recruiting maltreated children and adolescents, an effect of trauma onset on victims’ explicit emotion recognition was established [9]. In this context, it appears particularly interesting to extend previous researches conducted on street-boys, evaluating in a younger population the effects of high level of maltreatment on the explicit recognition of facial expressions of emotions.

To this aim a sample of street-children, and an age-matched control group, were submitted to a forced-choice facial expressions recognition task. If a recognition bias for angry facial expression will be present, anger label should be the most used (high Anger Tendency rate) and the most erroneously over-attributed label (high frequency of Anger false-alarms) with respect to the other alternative labels. Furthermore, if maltreatment exposure induces specific adjustments in the explicit recognition of facial expressions of emotions also in a children population, similarly to what evidenced among adolescents [21], the recognition bias for angry facial expressions should be significantly more pronounced among street-children than among age-matched controls.

It has been demonstrated that the individual cognitive level plays a key role in the correct identification of facial expressions of emotions [23], most likely because it measures fluid and crystallized abilities that are shaped by both neurological development and prior learning experiences [24]. Refinement of fluid and crystallized abilities corresponds with a developmental trend of improved emotions recognition from childhood [25], through adolescence [26], and into adulthood [27]. Thus, individual differences in cognitive level may have differential impact on one’s ability to recognize facial expressions of emotions in others. Furthermore, naming skills are involved in behavioral tasks which require an explicit and verbal identification of visual stimuli. Taking into account these considerations, participants’ cognitive level and naming skills were measured by means of validated tests. Between-groups differences, as well as the influence of these variables on participants’ behavioral performance were investigated. If childhood experiences of maltreatment influence the explicit recognition of facial expressions of emotions, inducing a bias for anger recognition, it should be independent from individual cognitive level and naming skills.

## Materials and Methods

### Participants

A total of 64 Sierra Leonean children were recruited for the study. Two participants were excluded from the analyses due to difficulties in task execution, resulting in a final sample of 62 participants. Of these 31 were street-children (STch) and 31 were control children (Con) who had never been street-children, who lived with their parents or close relatives and who regularly attended to school. The sample size exceeded the minimum amount required (n.36) estimated by means of statistical power analysis (a priori sample size n. evaluated for 1-ß = 0.95, α = 0.05 and effect size = 0.25). The sampling was suspended when two sex-balanced groups of enough size were obtained. Street-children were recruited through local organizations active in the socio-sanitary assistance to homeless children. Principally street-children involved in the study came directly from the street or from schools enrolling street-children. Controls were recruited from the community thanks to the collaboration of local organizations and public schools. The general purposes and procedures of the study were explained by local social-workers to volunteers, and their legal guardians, before written informed consents collection. All participants assisted by guardians, filled an anamnestic semi-structured interview through which their demographic information (i.e., sex, age, schooling, first language), life conditions (i.e., amount of time spent on the street, street activities, housing details, access to basic needs and health care, medical history), critical life events (i.e., sexual and physical abuses, mourning) and their socio-economic status (SES; i.e., family income, caregivers’ employment and schooling) were obtained. Partial or unclear information was completed and checked thanks to the collaboration of sanitary, educational and charitable institutions. Children for which data had not a reliable confirmation were not recruited for the study. Participants’ age was balanced between STch and Con (STch: mean = 7.65 years ± 1.68, SE 0.30, age-range = 5–10 years, median = 8; Con: mean = 7.77 years ± 1.78, SE 0.32, age-range = 4–12 years, median = 8) with no significant differences (t_60_ = 0.29; *p* = 0.77). Similarly, participants’ years of schooling (STch: mean = 2.55 years ± 1.31, SE 0.24, schooling-range = 1–6 years, median = 3; Con: mean = 2.45 years ± 1.26, SE 0.23, schooling-range = 1–6 years, median = 2) resulted no significantly different (t_60_ = 0.30; *p* = 0.77). A detailed demographic description of street-children and controls is noted in Table 1.

**Table 1:** Socio-demographic description of the samples.

		STch	Con
N. Tot		31	31
n. male		16	15
Age (years)		7.65 SE 0.30	7.77 SE 0.32
Schooling (years)		2.55 SE 0.24	2.45 SE 0.23
First Language (%)	Temne	61.29	9.67
	Mende	22.58	9.67
	Limba	9.68	35.48
	Krio	0	12.90
	English	0	16.12
	Other	6.45	16.13
Homeless children (%)		100	0
Daytime spent on the street (hours)		8.3 SE 0.26	2 SE 0.06
Night-time spent on the street (hours)		10.74 SE 0.11	0 SE 0.03
Street-activities (%)	Provide food and shelter	91	0
	Work	89	0
	Robberies	77	4.6
	Play	69.41	88
Health care coverage (%) ^a^		12	87.86
Access to basic needs (%) ^b^		20.15	96
Critical life events (%)	Physical Abuse	50	18.6
	Sexual Abuse	18.6	0
	Physical & Sexual Abuses	22	0
	Mourning	86.57	46.82
Presence of an Adult Caregiver (%)		2	100
Monthly family Income in Leones (%)	<200,000 Le	100	2.43
	200,000–500,000 Le	0	13.58
	500,000–700,000 Le	0	37.73
	>700,000 Le	0	17.64
Caregivers' Schooling (years)		-	5 SE 0.54
Caregivers' Employment (%)	Full-time salaried jobs ^c^	-	7.12
	Occasional job	-	23.15
	Trader	-	25.6
	Driver	-	12.69
	Artisan	-	16.02
	Miner	-	15.42

Street-children (STch) and controls (Con) socio-demographic characteristics. Numbers may not add to total due to missing data or rounding.

^a^–Health care coverage was defined as children’s access to preventive healthcare (i.e., vaccination, disease screening, malaria protection) and basic disease treatments (i.e., treatment of malaria, fever and diarrhea).

^b^–Access of basic needs was defined as children’s possibility to obtain adequate food, clean water, clothes and shelter.

^c–^Full-time salaried jobs comprehend physician, nurse, educator, employee, social worker.

### Standardized tests

In order to evaluate participants’ cognitive level and naming skills, Colored Progressive Matrices (CPM) [28] and Boston Naming Test (BNT) [29] were performed. CPM is a non-verbal test, measuring general cognitive abilities in terms of mental age, intellectual performance and non-verbal intelligence, designed for children aged 5^1/2^ through 11^1/2^ years of age. CPM requires non-verbal multiple choice responses to three sets of twelve matrices presented on a colored background. BNT assesses visual naming ability and word retrieval through 60 line drawings graded in difficulty and frequency. It is frequently administered to healthy children and adults. Tests selection was influenced by the lack of assessment instruments validated and applicable to west-African childhood population. Among tests evaluating cognitive performance, CPM was selected because, although not validated, normative values are reported in literature as it was already extensively used across a wide variety of settings in Africa [30]. BNT was chosen thanks to its quick and easy administration and because it is translated in many languages and commonly used in many countries.

The lack of validated and applicable scales on underage west-African population, as well as, the absence of effective nosographic investigations of psychiatric sequelae in non-West countries prevented the assessment of participants’ psychiatry conditions. In particular, considering our sample, the post-traumatic stress symptoms measurement by formal questionnaires was missing. To compensate for that limit participants’ electrocardiogram (ECG) was recorded for two minutes in a rest condition to extract participants’ heart rate (HR), a valid index of stress-related physiological response. A huge variety of studies demonstrated the presence of autonomic dysregulation among adults and children suffered from post-traumatic stress disorder (PTSD). Elevated HR [31–34] during rest conditions was attested also among full and sub-syndromal PTSD children [32] and even 7 years after trauma [33], and considered a valid physiological index of typical PTSD alterations in arousal and reactivity to external stimuli [35].

### Procedure

The experimental session took place in a quiet room and consisted in a forced-choice facial expressions recognition task [21]. Participants were asked to identify adults’ facial expressions of emotions choosing one of the four proposed labels (i.e., anger, fear, joy, sadness). Participants sat comfortably at a table, in front of a computer monitor (1024X768@75Hz). They were instructed to pay attention and to observe each stimulus for its entire duration. Each experimental trial started with the presentation of the question “you able du am?” (i.e. “Are you ready?”) on the PC monitor. After participants’ affirmative answer the experimenter pressed the spacebar to show the stimulus. This procedure was followed to ensure that participants’ attention was focused on stimuli presentation. Each stimulus was displayed once (64 total trials, 16 trials for each emotion: anger, fear, joy and sadness) in a random order. After each stimulus, with no time limit, participants were asked to identify which of the four alternative labels (anger, fear, joy, sadness) best described the facial expression of emotion displayed in the stimulus just shown. The four alternative labels were always visible and written in English and Krio on a sheet of paper. Participants’ answers were verbally expressed and transcribed by the experimenter. We preferred to avoid participants’ direct interaction with a response platform because of their unfamiliarity with electronic devices. The total duration of the forced-choice facial expressions recognition task was approximately 15 minutes, depending on participants’ answer time.

All participants were tested in the same location and using the same experimental setting. A local social-worker was always present to ensure that participants remained at ease, understood the instructions and to translate from English to Krio, if necessary. E-Prime 2.0 software (Psychology Software Tools, Inc.) was used to stimuli presentation.

The experimental protocol was approved by the Ethic Committee of the Ministry of Health and Sanitation of the Republic of Sierra Leone and it was in line with the Declaration of Helsinki 2013.

Stimuli employed in the forced-choice facial expressions recognition task were 64 videos obtained by the Montreal Set of Facial Displays of Emotion [36]) and already used in previous experiments conducted on adolescent [21] and adult African populations [37]. The stimuli were constructed by means of a face-morphing software (Squirlz Morph, http://www.xiberpix.net/SqirlzMorph.html), using one neutral facial expression as start image, and one emotional facial expression of the same actor, as end image. Among the facial expressions of emotions available in the Montreal Set of Facial Displays of Emotion database anger, fear, joy and sadness facial expressions of emotions were selected. Each video, lasting 3000 msec (15 fps; 800×560 pixels), showed the transition from a neutral facial expression to an emotional one (16 anger, 16 fear, 16 joy and 16 sadness). The Montreal Set of Facial Displays of Emotion images chosen to build the videos were selected pseudo-randomly from the Asian, African, Hispanic and Caucasian sets to include 16 instances of each of the chosen expressions (i.e., anger, fear, joy, sadness), balanced for gender and ethnic group.

### Statistical data analyses

In order to assess possible between-groups differences in cognitive level, naming skills and stress-related physiological response, three independent-sample t-tests (two-tailed) were performed on CPM score, BNT score and HR values comparing STch and Con groups. Significant between-groups difference in naming skills was better investigated evaluating if BNT-score was predicted by participants’ age, cognitive or educational levels. To this aim, age, CPM-score and years of schooling were included as predictors in two hierarchical regression analyses (forward-stepping), conducted independently for STch and Con, with BNT-score as dependent variable.

The presence of a recognition bias for angry facial expressions was assessed conducting a series of ANCOVAs separately on participants’ Tendency rate (percentage of use of each emotion label regardless of accuracy), General-false-alarms rate (percentage of incorrect use of each emotion label), Emotion-false-alarms rate (percentage of incorrect use of each emotion label calculated considering each emotion separately) and Accuracy rate (percentage of correct use of each emotion label). For each ANCOVA Group (STch, Con) was entered as between-factor; and Emotion (anger, fear, joy and sadness; or three of them in Emotion-false-alarms analyses) as within-factor. Participants’ BNT-score, CPM-score, years of schooling and age were entered as covariates.

A possible ethnicity effect on Accuracy rate was investigated performing an ANCOVA on participants’ Accuracy rate using Group (STch, Con) as between-factor and, as within-factors, Emotion (anger, fear, joy and sadness) and Ethnicity (Africa, Asian, Hispanic, Caucasian). Participants’ BNT-score, CPM-score, years of schooling and age were entered as covariates.

Accordingly to guideline [38] for all ANCOVA analyses, when the sphericity assumption was violated, Greenhouse-Geisser–correction was calculated and adjusted degrees of freedom (df), corrected p values, and epsilon values (Ɛ) reported. Whenever appropriated, significant between-groups and within-group differences were explored performing Bonferroni corrected t-tests (two-tailed). Partial eta square (ƞ^2^_p_) was calculated as effect size measure. Linear regression analyses were performed to deepened the significant covariate effects.

The possible influence of stress-related physiological response (assessed by HR measurement) on participants’ behavioral performance was underpinned by means of six linear regression analyses conducted separately for the two groups. HR was entered as predictor, whereas average Accuracy rate (percentage of correct responses), Anger Tendency rate (percentage of use of anger label regardless of accuracy) and Anger General-false-alarms rate (percentage of incorrect use of anger label) were included as dependent variables, time after time.

## Results

### Cognitive performance, naming skill and ECG variables

No significant between-groups difference was found in CPM-score (STch: 18.06 SE 0.70; Con: 17.97 SE 0.71; t_60_ = 0.097; *p* = 0.92) and HR (STch: 101.39 bmp SE 2.29; Con: 96.41 bmp SE 1.62; t_52_ = 1.66; *p* = 0.103) values. Otherwise, significant between-groups difference was found in BNT-score (STch: 11.03 SE 0.75; Con: 15.84 SE 1.17; t_60_ = -3.46; *p* < 0.005).

Regression analysis conducted on BNT-score of STch (R^2^ = 0.30; F_(1,29)_ = 12.51; *p* < 0.005) demonstrated that age is the only significant predictor (t = 3.54, β = 0.55, *p* < 0.005).

The same regression analysis conducted for Con (R^2^ = 0.48; F_(1,29)_ = 26.47; *p* < 0.001) revealed that years of schooling resulted the only significant predictor (t = 5.15, β = 0.69, *p* < 0.001).

### Response tendency

**Fig 1:**
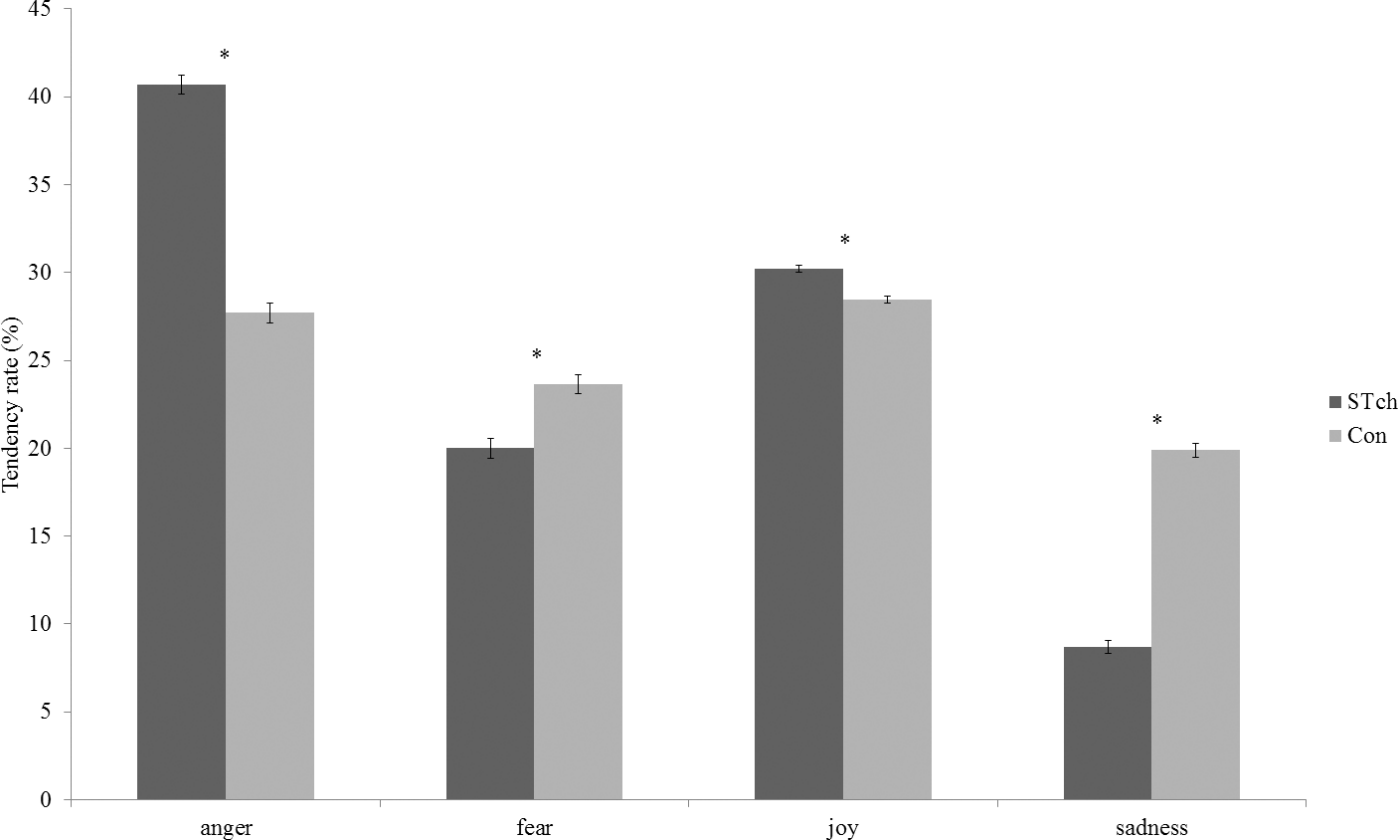
Response Tendency. Tendency rate for street-children (STch) and controls (Con). * = p < 0.005. Only between groups differences are shown. For differences within groups, see text. Error bars represent SE.

Mauchly’s test conducted on Tendency rate indicated that the assumption of sphericity had been violated (χ^2^_(5)_ = 71.15, *p* < 0.001), therefore df were adjusted using Greenhouse-Geisser correction (Ɛ = 0.63). The ANCOVA conducted on Tendency rate revealed that the factor Emotion was significant (F_1.9,105.3_ = 3.98; *p* < 0.05; ƞ^2^_p_ = 0.07), as well as the interaction of Emotion by Group (F_1.9,105.3_ = 5.55; *p* < 0.005; ƞ^2^_p_ = 0.09) (Fig 1). Bonferroni corrected t-tests (with α 0.05 = 0.005) conducted on Emotion by Group interaction revealed that STch used significantly more frequently the anger label than all other labels (anger *vs*. fear: t_60_ = 26.57, *p* < 0.0001; anger *vs*. joy: t_60_ = 17.78, *p* < 0.0001; anger *vs*. sadness: t_60_ = 47.25, *p* < 0.0001), whereas Con used that label more frequently only with respect to the other negative labels (anger *vs*. fear: t_60_ = 5.20, *p* < 0.0001; anger *vs*. joy: t_60_ = -1.28, *p* = 0.21; anger *vs*. sadness: t_60_ = 11.55, *p* < 0.0001) Furthermore, comparing the two groups, STch used significantly more frequently the anger (STch *vs*. Con: t_60_ = 16.42, *p* < 0.0001) and joy (STch *vs*. Con:t_60_ = 6.64, *p* < 0.0001) labels, and significantly less frequently fear (STch *vs*. Con: t_60_ = -4.79, *p* < 0.0001) and sadness (STch *vs*. Con: t_60_ = -20.72, *p* < 0.0001) labels than Con.

### General false alarms

**Fig 2:**
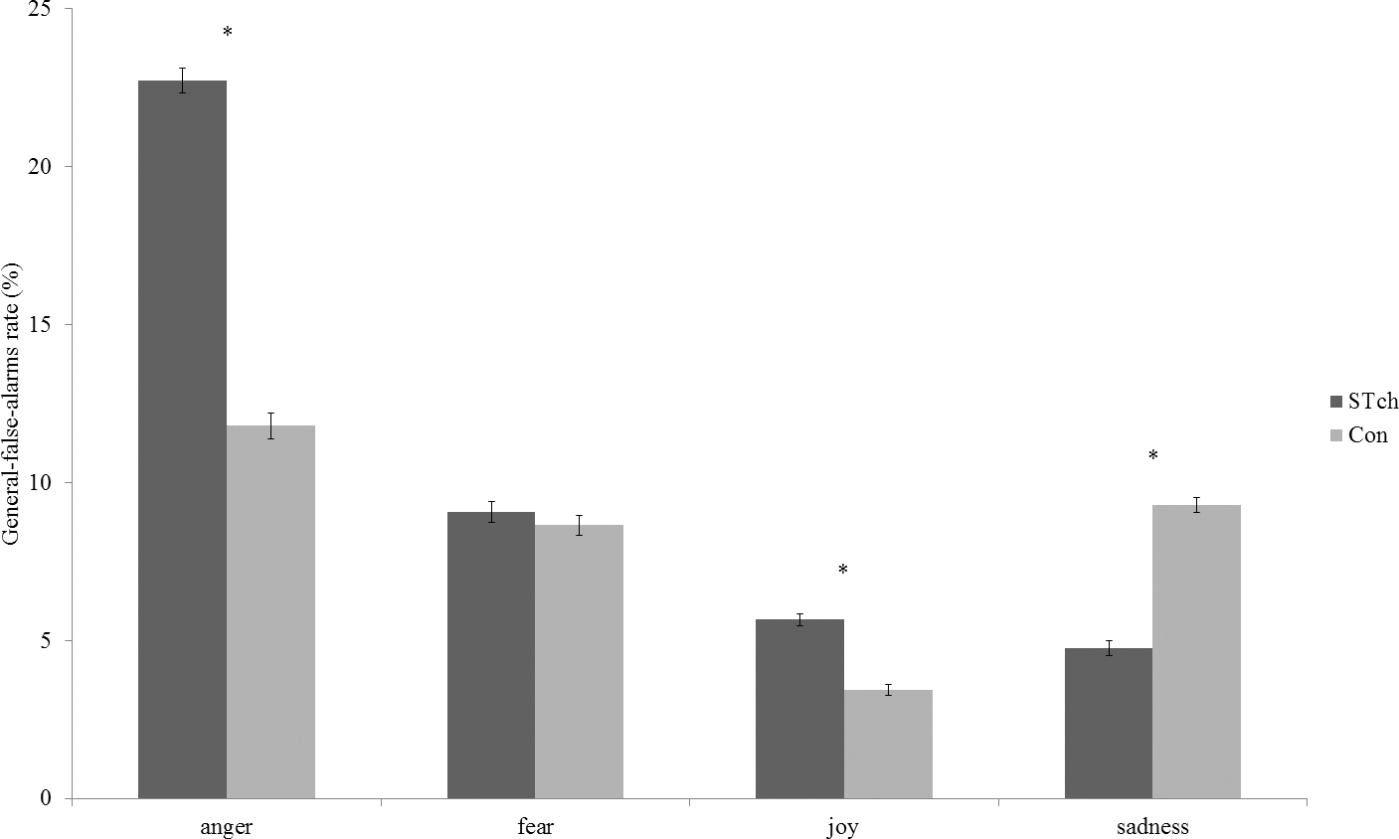
General-false-alarms. General-false-alarms rate for street-children (STch) and controls (Con). * = p < 0.004. Only differences between groups are shown. For differences inside each group, see text. Error bars represent SE.

Mauchly’s test conducted on General-false-alarms rate identified a sphericity violation (χ^2^_(5)_ = 38.64, *p* < 0.0001), hence df were adjusted using Greenhouse-Geisser correction (Ɛ = 0.68). ANCOVA performed on General-false-alarms rate revealed a significant effect of Group (F_1,56_ = 11.96 *p* < 0.005; ƞ^2^_p_ = 0.18) and a significant Emotion by Group interaction (F_2,114.90_ = 5.378 *p* < 0.005; ƞ^2^_p_ = 0.09) (Fig 2). Moreover, CPM-score, entered as covariate, resulted significant (F_1,56_ = 8.24 *p* < 0.05; ƞ^2^_p_ = 0.13), as well as the interaction Group by CPM-score (F_2,53_ = 3.74 *p* < 0.05; ƞ^2^_p_ = 0.12). Bonferroni corrected t-test (with α 0.05 = 0.004) performed on the main effect of Group showed that STch (10.56% SE 0.08) had a significantly higher General-false-alarms rate than Con (8.30% SE 0.08) (t_60_ = 20.62, *p* < 0.0001). Bonferroni corrected t-tests performed on the interaction Emotion by Group revealed that the wrongly use of the anger label was more frequent than the incorrect use of all other labels both for STch (anger *vs*. fear: t_60_ = 26.88, *p* < 0.0001; anger *vs*. joy: t_60_ = 39.02, *p* < 0.0001; anger *vs*. sadness: t_60_ = 38.68, *p* < 0.0001) and Con (anger *vs*. fear: t_60_ = 6.18, *p* < 0.0001; anger *vs*. joy: t_60_ = 19.11, *p* < 0.0001; anger *vs*. sadness: t_60_ = 5.39, *p* < 0.0001). Despite this similar trend, the two groups showed a significant different rate in the mistaken use of anger, joy and sadness labels. Bonferroni corrected t-tests showed that STch used more frequently the anger (STch *vs*. Con:t_60_ = 19.47, *p* < 0.0001) and joy (STch *vs*. Con:t_60_ = 8.56, *p* < 0.0001) labels, and less frequently the sadness label (STch *vs*. Con:t_60_ = -4.53, *p* < 0.0001) than Con.

**Fig 3:**
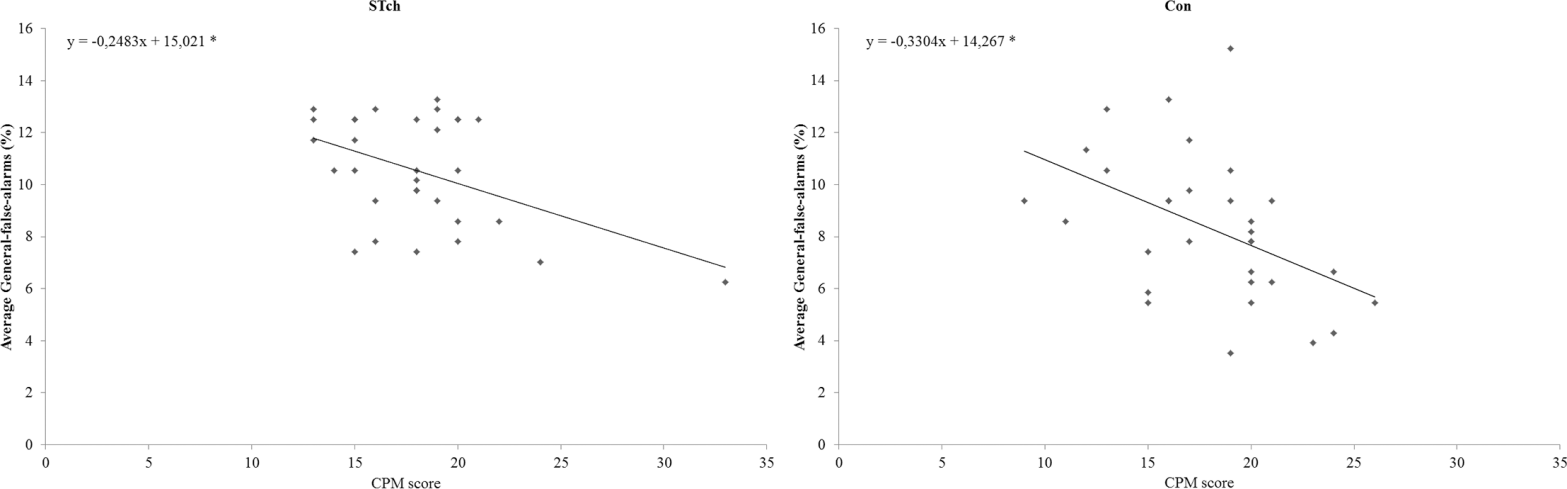
CPM-score predicts average General-false-alarms rate. Plots of CMP-score versus average General-false-alarms for street-children (STch) and controls (Con). * = p < 0.05.

To investigate the significant Group by CPM-score interaction two linear regression analyses were conducted separately for the two experimental groups, using the average General-false-alarms rate as dependent variable and CPM-score as predictor (Fig 3). Both regression analyses (STch: R^2^ = 0.21; F_(1,29)_ = 7.81; *p* < 0.05; Con: R^2^ = 0.22; F_(1,29)_ = 8; *p* < 0.05) demonstrated that CPM-score inversely predicted the average General-false-alarms rate (STch: t = -2.79, β = -0.46, 95% CI = -0.43 to -0.07, *p* < 0.05; Con: t = -2.83, x = -0.46, 95% CI = -0.57 to -0.09, *p* < 0.05) in both groups. The regression coefficients of the two groups resulted not significantly different, as reflected by their CIs.

### Emotion false alarms

#### Anger emotion

ANCOVA results revealed a significant interaction Emotion by BNT-score. Three linear regression analyses were conducted, separately for each emotion label, using the Anger Emotion-false-alarms rate recorded for each label as dependent variable, and the BNT-score as predictor. Regardless of group membership, BNT-score predicted the erroneous use of sadness label in response to angry facial expression presentation (R^2^ = 0.22; F_(1,60)_ = 16.95; *p* < 0.001; t = 4.12, β = 0.47, 95% CI = 0.25 to 0.72; *p* < 0.001). On the contrary, BNT-score did not predict the erroneous uses of fear (R^2^ = 0.03; F_(1,60)_ = 2.14; 95% CI = -0.45 to 0.07; *p* = 0.15) and joy (R^2^ = 0.001; F_(1,60)_ = 0.06; 95% CI = -0.07 to 0.05; *p* = 0.81) labels in response to angry facial expression presentation.

#### Fear emotion

**Fig 4:**
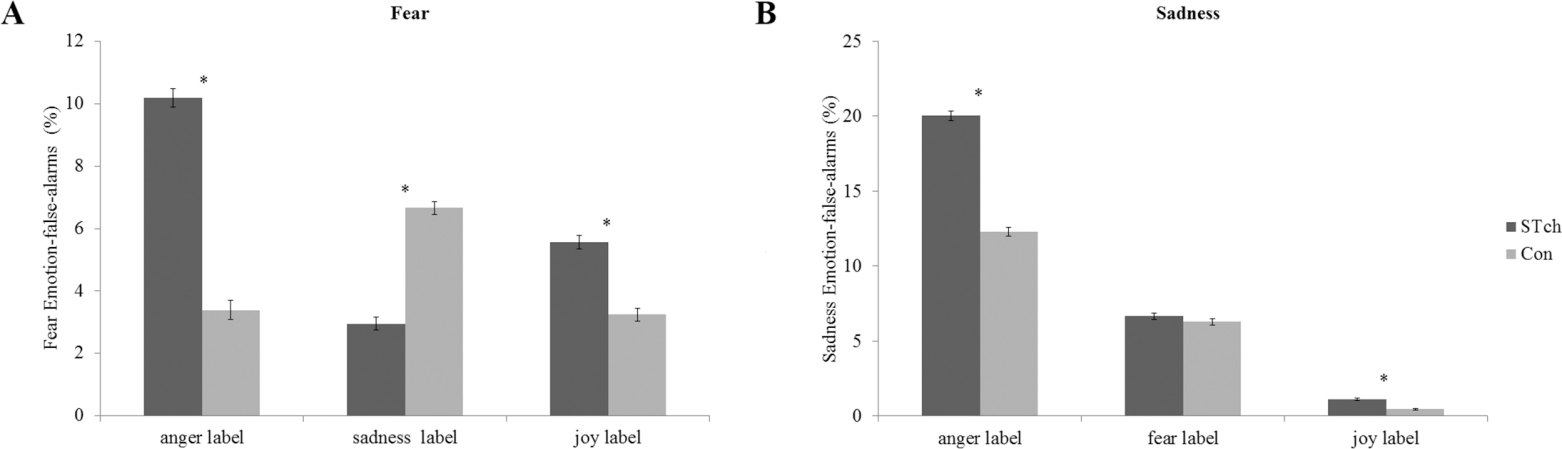
Fear and Sadness Emotion-false-alarms. A) Fear Emotion-false-alarms for street-children (STch) and controls (Con). * = p<0.007. B) Sadness Emotion false alarms for street-children (STch) and controls (Con). * = p<0.006. Both panels show only differences between groups. For differences inside each group, see text. In both panels error bars represent SE.

ANCOVA results revealed a significant interaction Emotion by Group (F_2,112_ = 5.90 *p* < 0.005; ƞ^2^_p_ = 0.09) (Fig 4, panel-A). Moreover, CPM-score, entered as covariate, resulted significant (F_1,56_ = 4.38 *p* < 0.05; ƞ^2^_p_ = 0.07). Bonferroni corrected t-tests (with α 0.05 = 0.007) performed on the interaction Emotion by Group showed that, considering STch participants, the incorrect use of anger label was significantly higher when compared with the mistaken use of the other two possible wrong labels (anger *vs*. joy: t_60_ = 12.47, *p* < 0.0001; anger *vs*. sadness: t_60_ = 19.56, *p* < 0.0001). Differently, evaluating Con participants, the use of anger label resulted significantly lower with respect to the inaccurate use of sadness label (anger *vs*. sadness: t_60_ = -8.84, *p* < 0.0001). Comparing the mistaken performance of the two groups in response to fear facial expressions presentation, Bonferroni corrected t-tests evidenced that STch used more frequently anger (STch *vs*.Con:t_60_ = 15.81, *p* < 0.0001) and joy labels (STch *vs*. Con:t_60_ = 7.73, *p* < 0.0001), and less frequently sadness label (STch *vs*. Con:t_60_ = -12.45, *p* < 0.0001) than Con.

**Fig 5:**
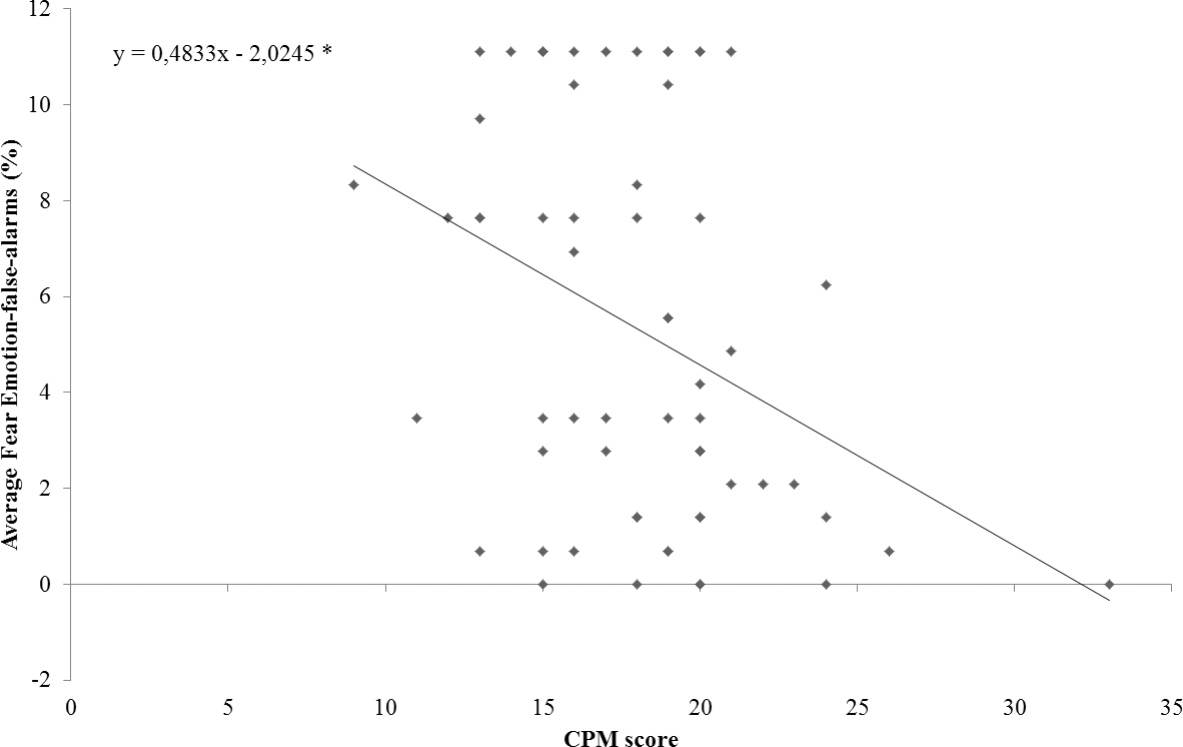
CPM-score predicts average Fear Emotion-false-alarms. Plot of participants’ CPM-score versus average Fear Emotion-false-alarms. * = p < 0.05.

To investigate the significant effect of CPM-score, a linear regressions (Fig 5) was conducted using the average Fear Emotion-false-alarms rate as dependent variable and CPM-score as predictor. Regression analysis demonstrated that, regardless of group membership, CPM-score inversely predicted the average Fear Emotion-false-alarms rate (R^2^ = 0.13; F_(1,60)_ = 8.88; *p* < 0.005; t = -2.98, β = -0.36, 95% CI = -0.63 to -0.12; *p* < 0.005).

#### Sadness emotion

Mauchly’s test conducted on Sadness Emotion-false-alarms rate showed a violation of sphericity assumption (χ^2^_(2)_ = 54,40, *p* < 0.001), therefore df were adjusted following Greenhouse-Geisser correction (Ɛ = 0.61). ANCOVA results showed that the factor Group (F_1,56_ = 15.60 *p* < 0.0001; ƞ^2^_p_ = 0.22) and the factor Emotion were significant (F_1.2,68.79_ = 5.04 *p* < 0.05; ƞ^2^_p_ = 0.08), as well as the interaction Emotion by Group (F_1.2, 68.79_ = 3.80 *p* < 0.05; ƞ^2^_p_ = 0.06) (Fig 4, panel-B). Bonferroni corrected t-test (with α 0.05 = 0.006) performed on the main effect of Group showed that STch (9.26% SE 0.09) had a significantly higher Sadness Emotion-false-alarms rate than Con (6.33% SE 0.09) (t_60_ = 23.55, *p* < 0.0001). Bonferroni corrected t-tests executed on the interaction Emotion by Group showed that both STch (anger *vs*. fear: t_60_ = 34.81, *p* <0.0001; anger *vs*. joy: t_60_ = 59.42, *p* <0.0001) and Con (anger *vs*. fear: t_60_ = 15.65, *p* < 0.0001; anger *vs*. joy: t_60_ = 37.19, *p* < 0.0001) erroneously used anger label significantly more frequently than the other two possible wrong labels. Comparing the mistaken performance of the two groups, Bonferroni corrected t-tests demonstrated that STch used more frequently anger (STch *vs*. Con:t_60_ = 17.57, *p* <0.0001) and joy (STch *vs*. Con:t_60_ = 7.24, *p* <0.0001) labels than Con. No significant difference was found for the mistaken use of fear label (STch *vs*. Con:t_60_ = 0.04, *p* = 0.97).

### Accuracy rate

**Fig 6:**
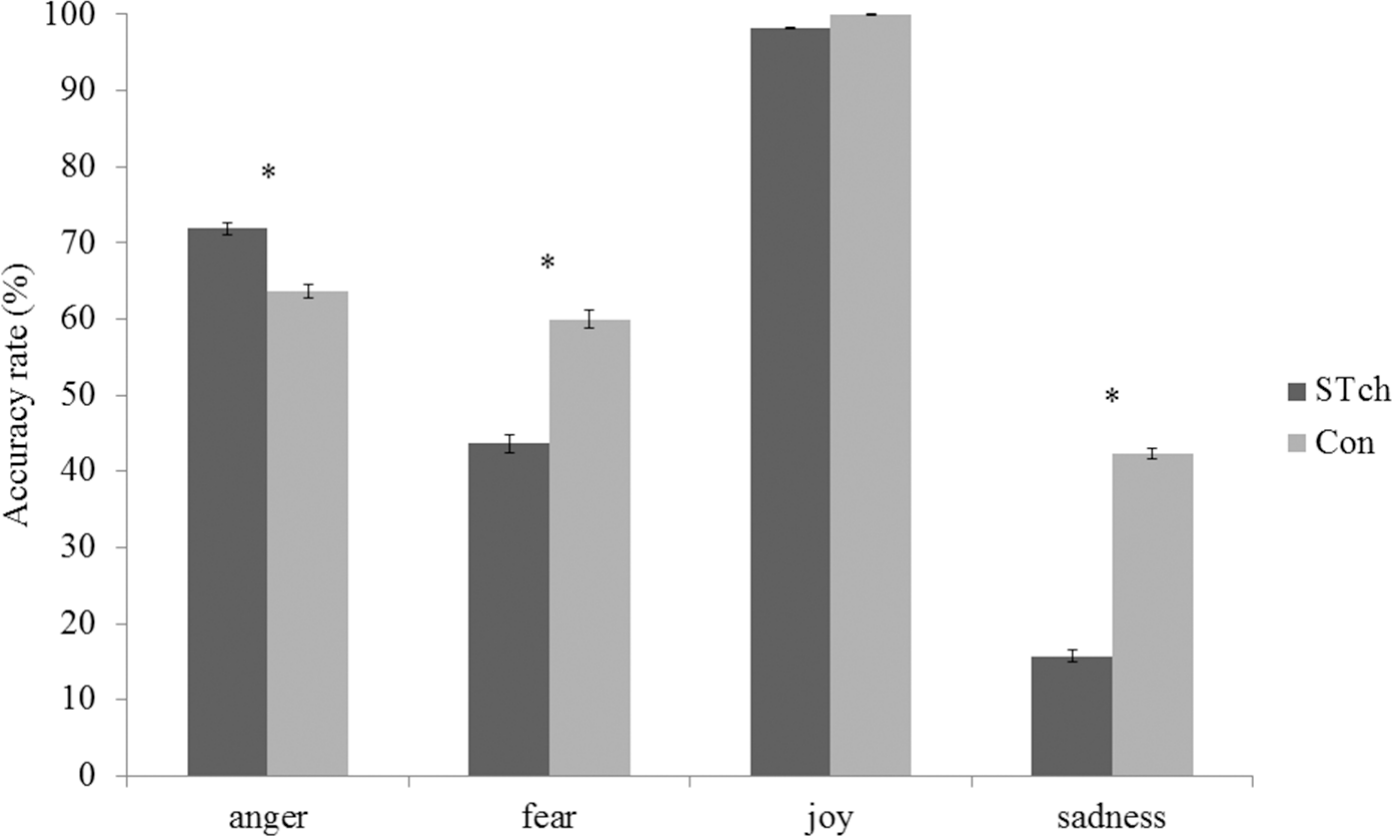
Accuracy rate. Accuracy rate for street-children (STch) and controls (Con). * = p < 0.004. Only differences between groups are shown. For differences inside each group, see text. Error bars represent SE.

Mauchly’s test conducted on Accuracy rate showed a violation of sphericity assumption (χ^2^_(5)_ = 75.95, *p* < 0.001), hence df were adjusted using Greenhouse-Geisser correction (Ɛ = 0.67). ANCOVA executed on Accuracy rate revealed a significant effect of the factors Group (F_1,56_ = 12.71 *p* < 0.005; ƞ^2^_p_ = 0.18) and Emotion (F_2.02,112.96_ = 10.47 *p* < 0.0001; ƞ^2^_p_ = 0.16), as well as of the interaction of Emotion by Group (F_2.02,112.96_ = 4.37 *p* < 0.05; ƞ^2^_p_ = 0.7) (Fig 6). Moreover, CPM-score (F_1,56_ = 9.33 *p* < 0.005; ƞ^2^_p_ = 0.14) and the interaction Group by CPM-score resulted significant (F_2,53_ = 4.29 *p* < 0.05; ƞ^2^_p_ = 0.14). Bonferroni corrected t-test (with α 0.05 = 0.004) performed on the main effect of Group showed that STch (57.35% SE 0.30) had significantly lower Accuracy rate than Con (66.49% SE 0.30) (t_60_ = -21.25, *p* < 0.0001). Moreover, Bonferroni corrected t-tests executed on the interaction Emotion by Group revealed that, considering STch, anger was significantly better recognized than sadness and fear facial expressions, but less identified than joy facial expressions (anger *vs*. fear: t_60_ = 19.65, *p* < 0.0001; anger *vs*. joy: t_60_ = -30.70, *p* < 0.0001; anger *vs*. sadness: t_60_ = 48.59, *p* < 0.0001). In the case of Con participants, angry facial expressions were better recognized only than sadness facial expressions, and less identified than joy expressions (anger *vs*. fear: t_60_ = 2.76, *p* = 0.007; anger *vs*. joy: t_60_ = -41.99, *p* < 0.0001; anger *vs*. sadness: t_60_ = 18.40, *p* < 0.0001). Comparing the two groups, STch recognized significantly less the facial expressions of fear (STch *vs*. Con:t_60_ = -10.04, *p* < 0.0001) and sadness (STch *vs*. Con:t_60_ = -24.44, *p* < 0.0001) than Con. Opposite, STch recognized significantly more angry facial expressions (STch *vs*. Con:t_60_ = 6.79, *p* < 0.0001) than Con.

**Fig 7:**
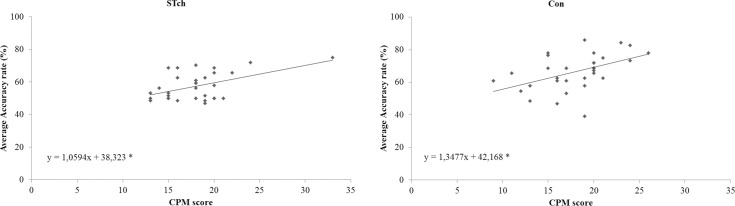
CPM-score predicts average Accuracy rate. Plots of CMP-score versus average Accuracy rate for street-children (STch) and controls (Con). * = p < 0.05.

To investigate the significant interaction Group by CPM-score two linear regressions were conducted (Fig 7), separately for the two experimental groups, using the average Accuracy rate as dependent variable and the CPM-score as predictor. Regression analyses demonstrated that CPM-score directly predicted the average Accuracy rate of both STch (R^2^ = 0.24; F_(1,29)_ = 9.26; *p* < 0.05; t = 3.04, β = 0.49, 95% CI = 0.35 to 1.77; *p* < 0.05) and Con groups (R^2^ = 0.23; F_(1,29)_ = 8.54; *p* < 0.05; t = 2.92, β = 0.48, 95% CI = 0.40 to 2.29, *p* < 0.05). The regression coefficients of the two groups were not significantly different, as reflected by their CIs.

#### Stimuli ethnic group and Accuracy rate

Mauchly’s test conducted on Accuracy rate showed a violation of sphericity assumption for the factor Emotion (χ^2^_(5)_ = 73.51, *p* < 0.001) and the interaction Emotion by Ethnicity (χ^2^_(44)_ = 126.23, *p* < 0.001), hence df were adjusted using Greenhouse-Geisser correction (Ɛ = 0.64; Ɛ interaction = 0.71). ANCOVA results revealed a significant interaction Ethnicity by CPM-score (F_2.97,6.05_ = 5.10 *p* < 0.05; ƞ^2^_p_ = 0.08). No other effects resulted significant.

To investigate the significant interaction four linear regression analyses were conducted including as dependent measures the average Accuracy rate of each Ethnic-stimuli-group and, as predictor, participants’ CPM-score. Results demonstrated that, regardless of participants’ group, CPM-score directly predicted the average Accuracy rate of African-stimuli-group (R^2^ = 0.14; F_(1,61)_ = 9.61; *p* < 0.005; t = 3.1, β = 0.37, 95% CI = 0.07 to 0.34, *p* < 0.05).

### Stress-related physiological response and behavioral performance

**Fig 8:**
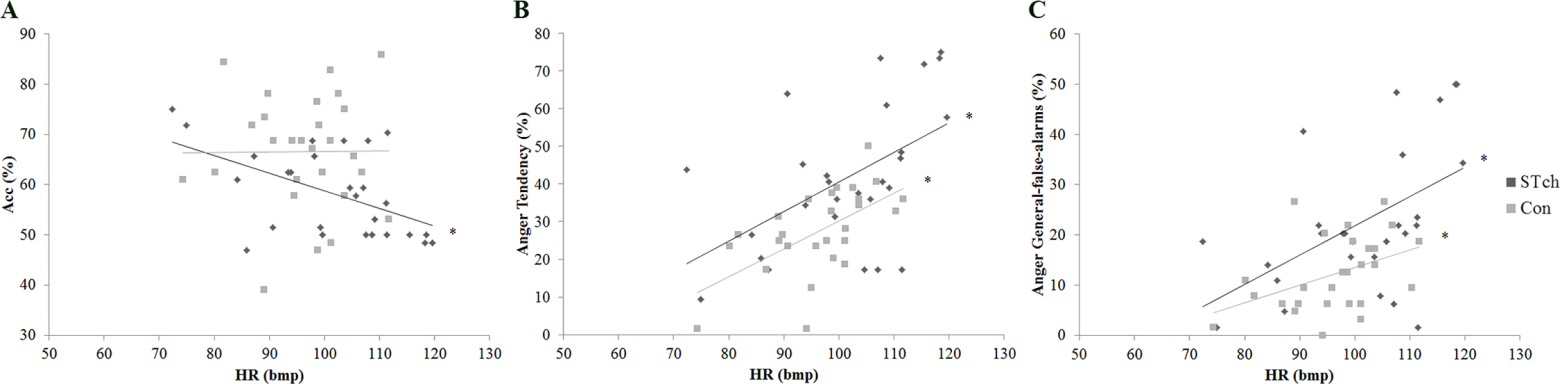
HR predicts behavioral performance. A) Plot of HR versus average Accuracy rate for street-children (STch) and controls (Con); B) Plot of HR versus Anger Tendency rate for street-children (STch) and controls (Con); C) Plot of HR versus Anger General-false-alarms rate for street-children (STch) and controls (Con). * = p < 0.01.

Regression analysis demonstrated that HR inversely predicted the average Accuracy rate of STch group (R^2^ = 0.27; F_(1,26)_ = 9.15; *p* < 0.01; t = -3.02, β = -0.52, 95% CI = -0.57 to -0.11). Differently, the same regression analysis conducted for Con group evidenced that HR was not a significant predictor (R^2^ = 0.01; F_(1,26)_ = 0.002; *p* = 0.96; t = 0.04, β = 0.01, 95% CI = -0.50 to 0.52) (Fig 8, panel-A).

Furthermore, both for STch and Con groups, regression analyses demonstrated that HR directly predicted their Anger Tendency rate (STch: R^2^ = 0.27; F_(1,26)_ = 9.22; *p* < 0.01; t = 3.04, β = 0.52, 95% CI = 0.26 to 1.34; Con: R^2^ = 0.35; F_(1,26)_ = 13.69; *p* < 0.005; t = 3.70, β = 0.59, 95% CI = 0.31 to 1.08) (Fig 8, panel-B) and Anger General-false-alarms rate (STch: R^2^ = 0.26; F_(1,26)_ = 8.88; *p* < 0.01; t = 2.98, β = 0.51, 95% CI = 0.18 to 0.97; Con: R^2^ = 0.18; F_(1,26)_ = 5.52; *p* < 0.05; t = 2.35, β = 0.42, 95% CI = 0.41 to 0.62) (Fig 8, panel-C).

## Discussion

The aim of the present study was to extend previous research [21], investigating the explicit recognition of facial expressions of emotions in a children population exposed to maltreatment. Two groups, one composed of street-children and one consisting of age-matched controls, performed a forced-choice facial expressions recognition task. Results demonstrated that both groups tended to use anger label more frequently than all other alternative labels (high Anger Tendency rate). Consequently, both groups over-attribute anger label to other negative facial expressions when they were incorrectly recognized (high Anger false-alarms rate). Relevant to the aim of the present study, anger over-attribution was significantly more pronounced among street-children than among controls, demonstrating the presence of the expected stronger anger recognition bias among street-children than controls. In fact, the analyses conducted on Tendency rate and General-false-alarms rate, demonstrated that in both cases street-children used anger label significantly more frequently with respect to controls. Moreover, Emotion-false-alarms investigations evidenced that street-children showed an over-attribution of anger both to fear and sadness facial expressions, whereas controls exhibited that tendency only for sadness expressions. Sure enough, when controls incorrectly recognized fear facial expressions, sadness label was over-attributed instead of anger label. As expected, these response patterns contributed to a significantly lower street-children’s Accuracy rate in sadness and fear recognition, and to a higher street-children’s Accuracy rate in anger recognition with respect to controls.

Taken together these results demonstrate that the exposure to maltreatment during childhood, similarly to what previously attested in an adolescent sample [21], induces an alteration of the explicit recognition of negative emotion, under which facial expressions of negative emotions are wrongly recognized as angry facial expression. The presence of a similar, tough significantly minor, tendency to use anger label among controls could be justify by, at least, two hypotheses referring to the natural development of facial expressions recognition, on one hand, and to environmental adaptive processes, on the other hand. Previous studies demonstrated that, at an initial developmental level, when joy facial expressions were compared with multiple negative expressions (e.g., sadness, fear, anger, and disgust), the negative ones were easily confused with each other [7]. It is only at a later developmental stage that the categorization of negative facial expressions become more accurate, generally starting from angry facial expression [2]. A proper investigation of false-alarms distribution across age is still lacking in literature. Nevertheless, it can be hypothesized that, at least at an initial developmental stage, when children are forced to discriminate among multiple negative facial expressions, as occurred in the present forced-choice task, they tend to predominantly use the label of the most salient and better recognized negative emotion, that is anger. On the other hand, live in an extremely disadvantaged social environment could promote, at least among children, the saliency of aversive facial expressions like anger inducing a behavioral effect on the tendency to use anger label during facial expressions of emotions recognition. Following this assumption, controls’ behavioral performance could reflect an adjustment of facial expressions recognition skills driven by environmental factors. This hypothesis is supported by the evidence of significant linear relations between participants’ HR and their Anger Tendency rate and Anger General false-alarms rate. In other words, all participants–both street-children and controls–showed a higher tendency in the use of anger label when they presented higher HR, an index of the physiological reaction to stress and also associated to PTSD symptoms. These results strengthen the thesis according to which the recognition bias for angry facial expressions is a manifestation of a functional adaptive mechanism that tunes victim’s perceptive and attentive focus on salient environmental social stimuli [12,13] highlighting also a possible additive effect as a function of children’s stress exposition.

Beside this consideration, the significant higher street-children’s recognition bias for angry facial expressions suggests that childhood exposure to a very abusive and neglectful environment, as happen to street-children, may amplify children’s pre-existing bias towards the identification of specific negative facial expressions. Participants’ naming skills and cognitive level influenced their global task performance (i.e., average Accuracy rate and average General-false-alarms rate) without affecting the recognition bias for angry facial expressions, as expected. Deficits in intellectual functioning were noted among maltreated children [39], which could extend throughout the life course [40] and potentially limit one’s ability to recognize emotions across developmental stages. Coherently, recent evidence demonstrated that maltreated females with lower levels of intellectual functioning were least accurate in identifying facial expressions of emotions, whereas those with higher levels of intellectual functioning performed as well as non-maltreated females [41]. In the present study, a significant between-groups difference in cognitive level was not found, whereas it was demonstrated that CPM-score influenced participants’ Accuracy rate especially for stimuli belonging to the same ethnic group of participants. The absence of CPM-score influence on General-false-alarms and Emotion-false-alarms rates extends previous researches demonstrating that the recognition bias for angry facial expressions is not influenced by victims’ cognitive performance.

Differently, participants’ naming skills, regardless of group membership, directly predicted only the erroneous use of sadness label during angry facial expressions presentation. Being sadness the less used label, it could be possible that higher naming skills facilitate its use in uncertain conditions in which the over-recognition of anger is not possible.

The between-groups difference in naming skills might be attributed to disparity in the quality of education of the two groups. Previous studies demonstrated that, BNT-score was related with years of schooling [42–44]. In the present study only controls’ BNT-score was predicted by educational level. On the other hand, street-children’s BNT-score was predicted by age. These results could be explained by considering the different socio-economic status of the two groups. Although the two groups had the same years of schooling, controls attended school regularly and were occupied in study even outside the school, whereas street-children were unable to engage themselves regularly in school. Moreover, controls attended a fee-paying school not available to street-children whom frequented a free school managed by volunteer teachers.

In conclusion, the present study demonstrates for the first time the presence of a recognition bias for angry facial expressions among street-children exposed to maltreatment. The presence of a similar tendency, although significantly less pronounced, among controls suggests that child maltreatment amplifies a children’s “pre-existing bias” for anger labeling in emotion recognition task probably provoked by extremely disadvantaged social environment in which all participants lived. This conclusion increases the need of a systematic and deep investigation of non-West countries psychological and psychiatric conditions especially among underage population. The recognition bias for angry facial expressions appears to be independent from victims’ age, cognitive and educational levels and from their naming skills.

Further researches have to explore whether, beside explicit recognition deficit, children exposed to maltreatment experiences manifest also altered implicit processes associated to facial expressions recognition, like automatic facial mimicry and autonomic regulation of social behaviors.

Some limitations of the present study should be highlighted. First, even if the effect of some influencing variables (i.e., participants’ age, sex, schooling, BNT score, CPM score) were controlled by sampling procedure and statistical analyses, other untestable factors could influence participants’ performance. Among others, the lack of any validated and applicable scales on underage west-African population prevented the formal assessment of participants’ psychiatric sequelae and SES through the use of standardized questionnaires. Moreover, the employment of a forced-choice recognition task, expressly designed to highlight possible participants’ bias in the recognition of negative facial expressions [21] prevented the extension of our results to different tasks (e.g., non-verbal or implicit tasks), as well as to other here non-tested facial expressions of emotions. For example, the inclusion of more than one positive facial expressions can shed light on potential bias among positive facial expressions recognition. Further studies, employing different tasks and stimuli, will fill this gap. Finally, the absence of the expected participants’ age influence on Accuracy rate [45–47] suggests that the present task could be not sufficiently sensitive to detect age-related increment in accuracy rate when it is implemented on this peculiar population of children.

## Supporting Information

S1 Dataset Dataset of participants’ behavioral responses, heart rate values and scores in standardized tests. (XLSX)
